# Clavicular hook plate for acute high-grade acromioclavicular dislocation involving Rockwood type V: clinical and radiological outcomes and complications evaluation

**DOI:** 10.1007/s00264-022-05498-8

**Published:** 2022-07-19

**Authors:** Guoming Liu, Yanling Hu, Fagang Ye, Fuguo Huang, Tengbo Yu

**Affiliations:** 1 Department of Orthopedics, Affiliated Hospital of Qingdao University, Qingdao University, Qingdao, Shandong 266003 People’s Republic of China; 2 Department of Orthopedic Surgery, West China Hospital, Sichuan University, Chengdu, Sichuan 610041 People’s Republic of China

**Keywords:** Acromioclavicular joint, Hook plate, Osteolysis, Subacromial impingement, Outcome

## Abstract

**Background:**

The surgical treatment of high-grade acromioclavicular joint dislocation remains a matter of debate. Clavicular hook plate internal fixation was widely used in the treatment of acromioclavicular dislocation because of its easy-to-master surgical technique. This study aimed to evaluate outcomes using hook plate fixation for acromioclavicular dislocation.

**Methods:**

A consecutive series of 57 patients with acute acromioclavicular joint dislocation involving Rockwood type V were treated between November 2013 and September 2019 using hook plate fixation. The functional outcomes (using the visual analogue score, Constant-Murley score, and University of California Los Angeles score), the quality of surgical reduction (using the coracoclavicular distance), and post-operative complications were assessed with about 46 months of follow-up.

**Results:**

The mean Constant-Murley score increased from 72.6 before surgery to 87.6 at final follow-up. The mean University of California Los Angeles score was 14.1 pre-operatively and 31.6 at final follow-up. Meanwhile, the visual analogue scores were significantly reduced from 3.4 pre-operatively to 1.3 post-operatively. The coracoclavicular distance decreased from 19.4 mm pre-operatively to 10.9 mm at the last follow-up. Post-operative functional and radiological outcomes were significantly improved compared with pre-operative outcomes (*P* < 0.01). The overall excellent and good result was 35.1% (20/57) and 54.1% (31/57), respectively. At follow-up, the overall complication rate was 15.8% (9/57) including subacromial impingement (three patients), acromial osteolysis (three patients), reduction loss (one patient), acromioclavicular joint osteoarthritis (one patient), and calcification (one patient).

**Conclusion:**

Hook plate fixation was a viable treatment approach, and achieved good clinical outcomes in the treatment of acute acromioclavicular dislocation involving V. But some complications of hook plate fixation should not be ignored.

Acute high-grade acromioclavicular dislocation (ACD) usually requires surgical treatment because of complete tear of acromioclavicular and coracoclavicular ligaments, with loss of stability [1–3]. The operative approach of Rockwood type III ACD has yielded good results, although it remains controversial [4, 5]. Restoration of the anatomic structure of the acromioclavicular joint (ACJ) eliminated the obvious deformity and simulated the native ACJ stiffness that might lead to more physiological stabilization [6].

Numerous surgical techniques have been recommended for treatment of acute ACD, including TightRope technique, hook plates, and single coracoclavicular suture fixation [7, 8]. So far, no standard technique has been established and a few complications have been described for these approaches. In a nationwide survey in Germany, the hook plate appeared to have become “standard therapy” for acute unstable acromioclavicular dislocations for the past few years [9].

Patients with unstable ACJ injuries managed with hook plates have shown reliable clinical outcomes [10]. However, it was reported that the clavicular hook plate caused subacromial shoulder impingement and rotator cuff lesion [11]. Previous studies reported that acromioclavicular fixation was more successful than coracoclavicular fixation [12]. It was reported that hook plate fixation for acromioclavicular joint disruptions provided biomechanically a stability similar to the native ACJ and allowed physiologic movement without pathological malformation [13]. The purpose of this study was to evaluate the functional and radiological outcomes of acute high-grade ACD treated with a hook plate.

## Patients and methods

### Study population

**Fig. 1:**
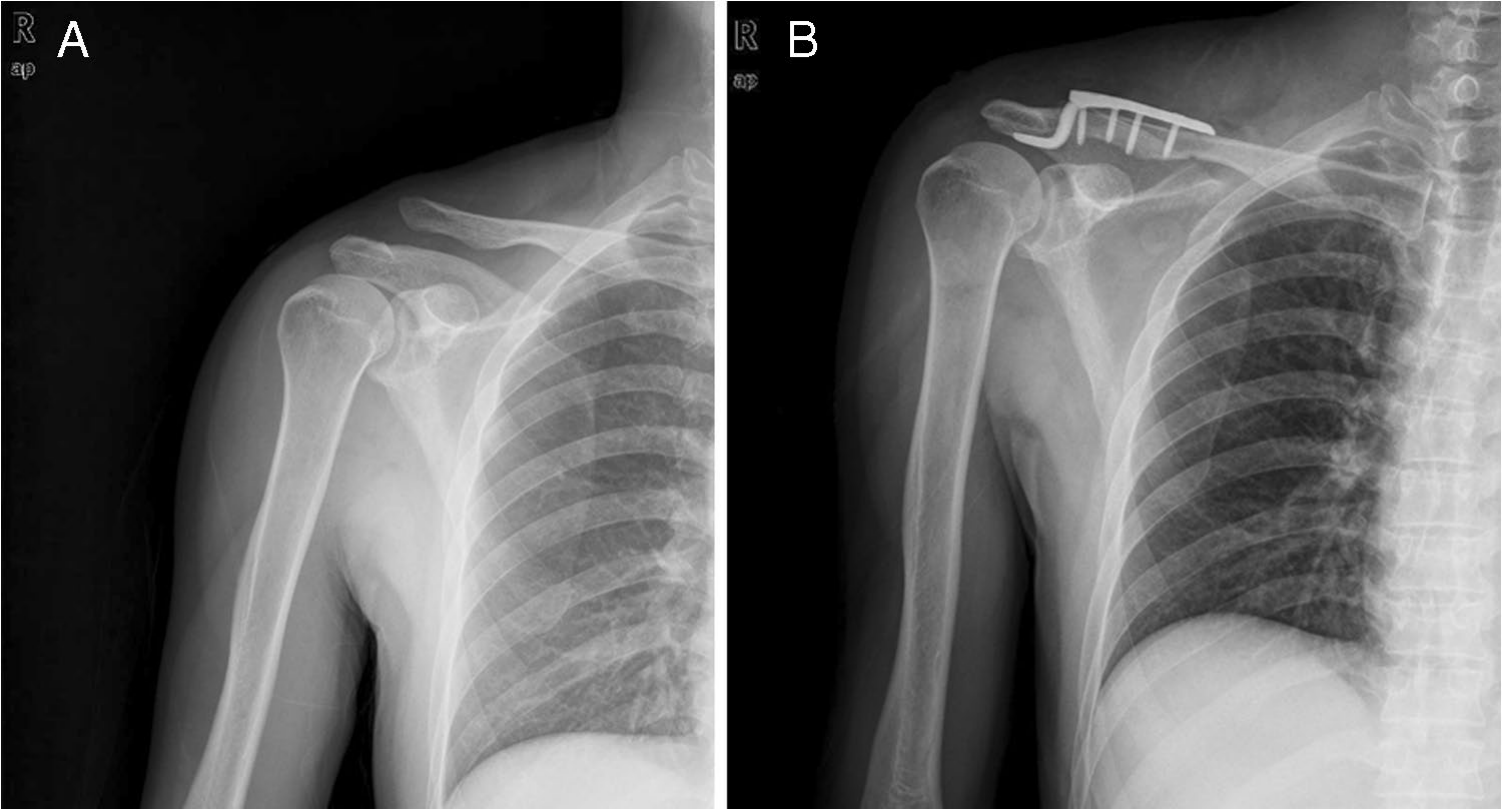
**A** Pre-operative radiograph showing grade V acromioclavicular dislocation. **B** Preoperative radiograph showing reduction of dislocation

A total of 92 patients with ACD were treated with a hook plate in our hospital from November 2013 to September 2019. All patients with an acute dislocation (injury within 3 weeks) and minimum clinical follow-up of 20 months were included. Only patients with Rockwood type V ACD met the inclusion criteria (Fig. 1A). Rockwood type V involved a complete rupture of the acromioclavicular ligament and coracoclavicular ligament as well as a more extensively rupture of the deltoid and trapezial fascia, manifested by an increase of more than 100% in the coracoclavicular distance radiologically [14]. All patients were older than 18 years and had no neurovascular injury of the shoulder joint. Patients with a history of shoulder stiffness, ACJ arthritis, and those who injured associated with ipsilateral scapular girdle fracture (clavicle fracture, scapular fracture, and humeral fracture) or received surgical intervention to the shoulder girdle were excluded. This study complied with the ethical standards of the Declaration of Helsinki and was approved by the ethics committee of the Affiliated Hospital of Qingdao University.

Overall, 64 patients met the inclusion criteria, and seven patients were lost for clinical and radiographic follow-up. Finally, 57 patients were included in this study. There were 39 men and 18 women with an average age of 38.8 years. The mechanisms of injury included nineteen traffic accidents (33%), twenty-seven falling down (47.4%), nine falling from a height (15.8%), and two bruise injuries by heavy object (3.5%). Operation was performed after an average delay of 1.9 days (range 1–5 days).

### Surgical technique

Operations were performed in the beach chair position under general anaesthesia. An incision 7 to 10 cm in length was made along the distal clavicle to acromion. Full-thickness subcutaneous flaps were made for exposure of the dislocated joint and the distal clavicle, and the dislocated joint was reduced and fixed using a suitable size hook plate. The hook plate was placed in posterior-inferior of the acromion without reconstruction of the coracoclavicular ligament. Reduction of dislocation was confirmed by intra-operative fluoroscopy (Fig. 1B). Finally, the incision was closed layer by layer.

After operation, a neck-wrist sling was used for protection and rehabilitation was started after two weeks. The hook plate was removed at 11.6 months after surgery followed by rehabilitation training. The mean follow-up period was 46.2 months with a range from 20 to 76 months.

### Clinical and radiographic assessment

Basic information of included patients was collected on age, gender, injury mechanism, time from injury to surgery, range of motion of the affected shoulder, and length of follow-up. Clinical assessment before and after surgery was performed using the visual analogue (VAS) score, Constant-Murley (CMS) score, and University of California Los Angeles (UCLA) scoring systems. The CMS score is a 100-point score, which consists of four parameters: pain (0–15 points); activity level (0–20 points), range of motion (0–40 points), and power (0–25 points) [15]. The UCLA score was evaluated by examining pain and function, as well as active forward flexion, strength of forward flexion, and satisfaction of the patient. The maximum score of UCLA scoring systems was 35 points [16]. Clinical outcomes were classified as excellent (91–100 points), good (81–90 points), fair (61–80 points), and poor (< 61 points) by the lmatani evaluation system [17].

Radiological evaluation was performed pre-operatively, post-operatively and at final follow-up, and post-operative plain radiographs were compared with pre-operative and contralateral plain radiographs. The images were analyzed and standardized to calculate the coracoclavicular distance (CCD) on anteroposterior views (CCD, height between the inferior border of the clavicle and the upper border of the coracoid process).

Acromioclavicular arthritis, acromial osteolysis, re-dislocation, CC calcifications, and implant-related complications (wound infection, soft tissue irritation, internal fixator loosen or breakage) were evaluated.

### Statistical methods

Date were given as mean ± standard deviation. ANOVA was used to evaluate significant differences in pre-operative, post-operative, and contralateral outcomes for continuous variables. A value of *P* less than 0.05 was considered as statistically significant. Statistical analysis was performed with SPSS version 16.0 software (SPSS, Chicago IL, USA).

## Results

The main characteristics of demographic and operative data are summarized in Table 1. All 57 patients were assessed for clinical outcomes using the Constant-Murley score and UCLA score criteria before surgery and at the end of follow-up. The mean Constant-Murley score increased from 72.6 points (range, 55–85) before surgery to 87.6 points (range, 75–100) at follow-up. There were statistically significant differences in Constant-Murley score between pre-operation and last follow-up. The mean UCLA score was 14.1 (range, 10–18) pre-operatively and 31.6 (range, 28–35) at final follow-up, which was a statistical difference between the time intervals. Meanwhile, VAS scores were significantly reduced from 3.4 (range, 1–7) pre-operatively to 1.3 (range, 0–4) post-operatively. The overall excellent and good result was 35.1% (20/57) and 54.1% (31/57), respectively. Detailed results of the functional outcome are shown in Table 2.

**Table 1:** Patients demographic and operative data overview

Parameter	Value
Male	39
Female	18
Age	38.8 (20–67)
Mechanism of injury	
Traffic accident	19 (33.3%)
Falling down	27 (47.4%)
Fall injury	9 (15.8%)
Bruise injury by heavy object	2 (3.5%)
Delay to surgery (days)	1.9 (1–5)
Operation time (min)	67.6 (35–110)
Blood loss (ml)	81.2 (40–215)
Follow-up (m)	46.2 (20–76)
Removal time (m)	11.6 (8–19)

**Table 2:** The outcomes and complications of ACD preoperatively and postoperatively using hook plate fixation (mean and standard deviation (SD))

Parameter	Preoperation	Postoperation	*P* value
Clinical evaluation			
CMS score	72.6 (6.6)	87.6 (5.4)	< 0.01
VAS score	3.4 (1.3)	1.3 (0.8)	< 0.01
UCLA score	14.1 (2.1)	31.6 (1.5)	< 0.01
Excellent		20 patients (35.1%)	
Good		31 patients (54.4%)	
Fair		6 patients (10.5%)	
Radiographic evaluation			
CCD (mm)	19.4 (4.0)	10.9 (1.7)	< 0.01
Complications			
Acromial osteolysis		3 patients (5.3%)	< 0.01
Subacromial impingement		3 patients (5.3%)	
ACJ osteoarthritis		1 patient (1.8%)	
Reduction loss		1 patient (1.8%)	
Calcification		1 patient (1.8%)	

*VAS score* visual analogue score, *CMS score* Constant-Murley score, *UCLA score* University of California Los Angeles score, *CCD* coracoclavicular distance

On the injured side of the shoulder, the CCD decreased from an average of 19.4 mm (range, 12.3–37.0 mm) pre-operatively to 10.9 mm (range 6.9–14.2 mm) at the last follow-up. The CCD was significantly improved after operation. There was a statistically significant difference between pre-operation and post-operation (*P* < 0.01). The average CCD on the contralateral side was 10.8 mm (range 5.7–16.3 mm), which was not significantly difference compared to the operative side (*P* = 0.813).

**Fig. 2:**
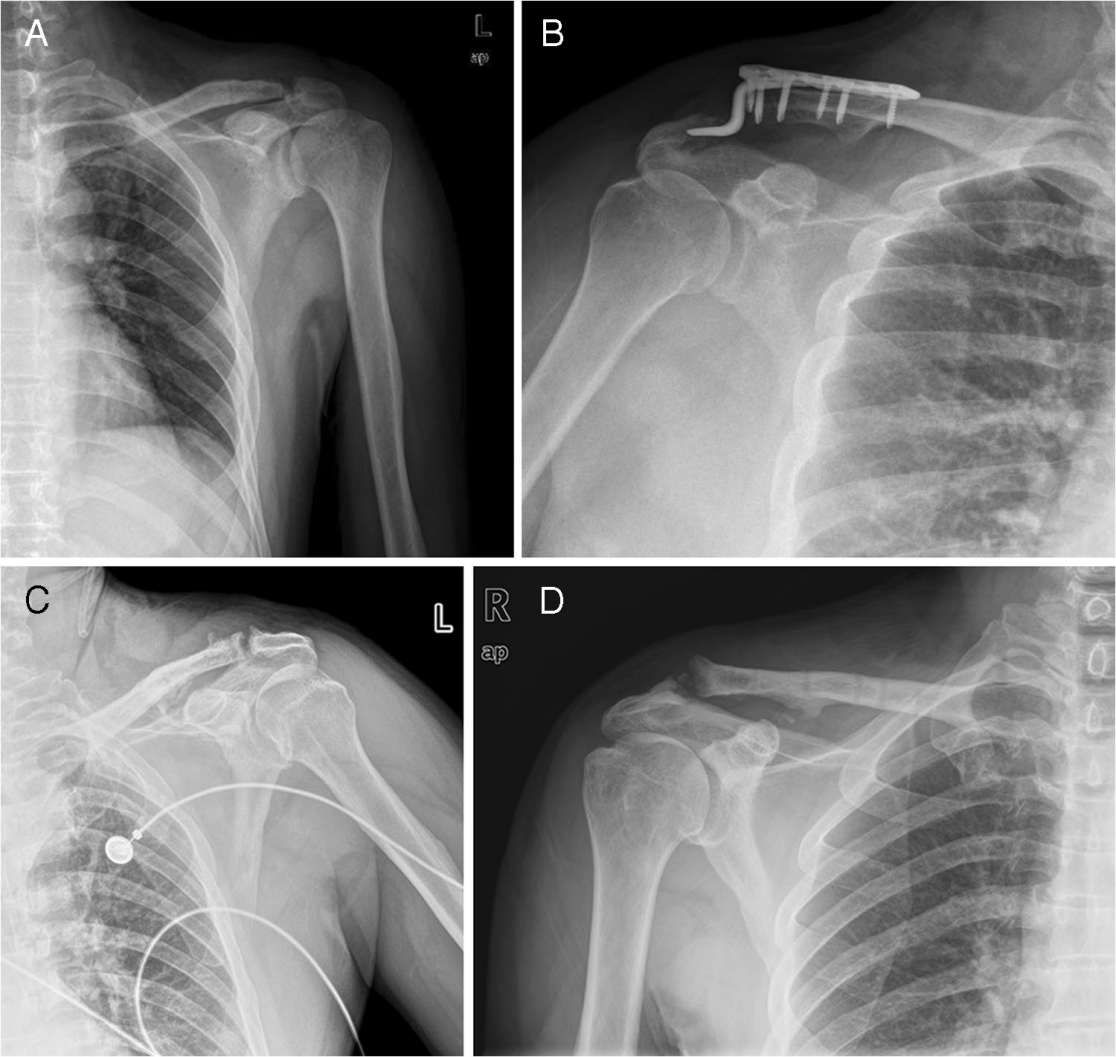
Acromial osteolysis (**A**), reduction loss (**B**), acromioclavicular osteoarthritis (**C**), and calcification (**D**) were shown on X ray after operation

The overall complication rate was 15.8% (9 complications). No wound infection occurred in any of the patients. No implant breakage occurred during the follow-up period. Three patients developed a subacromial impingement, and the symptoms disappeared after the plate was removed. Acromial osteolysis occurred in three patients by radiological assessment, which did not cause loss of motion after plate removal (Fig. 2A). Reduction loss was found in one patient during follow-up (Fig. 2B). We did not further perform reduction surgery due to the patient’s wishes. However, the patient did not have apparent discomfort after the removal of the plate. ACJ osteoarthritis (Fig. 2C) and (Fig. 2D) calcification were reported in one patient respectively. Mild pain was observed after the removal of hook plate, but it did not interfere with daily life. Detailed results of complications are shown in Table 2.

## Discussion

In this study, we demonstrated that good clinical outcomes could be achieved in the treatment of ACD using hook plate fixation, although some complications occurred. We thought that the hook plate fixation can be recommended as an alternative access to treat high-grade ACD involving V.

It was shown that surgical treatment for grade III–V ACD was superior to conservative treatment [18]. Currently, there are a number of surgical techniques described for operative treatment of ACJ dislocations [19]. No single surgical technique has clearly proven to be superior to other forms of fixation. Arirachakaran et al. published a systematic review and compared outcomes and complication rates of a suspensory loop fixation device (arthroscopic or open, TightRope or EndoButton, single or double) versus a hook plate in the treatment of ACD, and they confirmed that suspensory loop fixation had better shoulder function scores, lower post-operative pain, but higher complication rates compared with hook plate fixation [20].

Until now, clavicular hook plate internal fixation was widely used in the treatment of ACD because of its easy-to-master surgical technique [21]. Compared with arthroscopic ligament reconstruction, despite that the clavicular hook plate required a secondary procedure for removal, the internal fixation technique was simple and easy to perform [22]. Gunnar Jensen et al. found that outcomes of the clavicular hook plate were equal to the results by using the arthroscopic TightRope technique for the treatment of acute ACD [23]. Hamid Rahmatullah Bin Abd Razak et al. demonstrated that arthroscopic TightRope fixation had better short-term outcomes when compared to hook plate fixation for treating acute unstable ACD, but they also found that hook plate fixation had better forward flexion and strength during the first year after surgery [24]. In other studies, some researchers reported that hook plate fixation had better radiographic outcomes based on reduction maintenance compared with coracoclavicular ligament reconstruction [12, 25]. However, Clavert et al. confirmed that the complication rate of arthroscopic ligament reconstruction was high, up to 22%; the main complications include loss of reduction, adhesive capsulitis, coracoid fracture, and hardware pain, which affect the return to sports [26].

In our study, post-operative functional and radiological outcomes were significantly improved compared with pre-operative outcomes. The overall excellent and good results were 89.2% at the last follow-up. Our study coincided with that of Kienast et al., in which 89% of patients achieved excellent outcomes, and the overall complication rate was about 10% [27]. On the other side, Kumar et al. showed that clavicular hook plate fixation without coracoclavicular ligament reconstruction obtained good and excellent clinical results [28].

Several previous studies have demonstrated satisfactory functional outcomes of hook plate fixation for ACD. However, with the popularization of this application, various complications, such as acromial impingement, erosion of the acromion, and rotator cuff injury after hook plate removal, often gradually emerge [11]. Subacromial osteolysis was one of the common complications of ACD treated with the clavicular hook plate, and the complication rate was about 58% [29]. In our study, 10.6% of patients demonstrated acromial osteolysis and subacromial impingement. The slight discomfort disappeared after removal of the plate. One patient experienced reduction loss during the follow-up. No revision surgery was performed because of mild discomfort after the removal of the hook plate. We thought that exact positioning of the implant and accurate reduction of ACD reduced the incidence of complications in this study. Meanwhile, we believed that removal of the plate as early as possible after soft tissue healing was an effective way to reduce complications, although the optimal timing of plate removal remains controversial. Di Francesco et al. showed that re-dislocation occurred in 12% of patients in the treatment of acromioclavicular dislocation with a hook plate [30]. In addition, studies have demonstrated that the application of a hook plate had achieved good clinical outcomes despite loss of reduction [31, 32]. Although previous studies have shown that clinical outcomes were not directly correlated with radiographic findings based on osteolysis, the relatively high occurrence rate might suggest a disadvantage of hook plate fixation [33]. It was possible that different materials, angles, and depths of hook plates during fixation increased the stress between the plate and acromion, leading to osteolysis of the acromion [7, 10, 34]. The mismatch between anatomical morphology of acromion and existing design of the hook plate was considered to be one of the factors leading to subacromial impingement and osteolysis [35]. The possible reason for the osteolysis might be the stress concentration over the clavicle site after the fixation with the hook plate [35]. It was necessary to measure acromion height before surgery for reducing acromion impingement [36]. These studies indicated that it was necessary to use personalized hook plates for ACD to reduce the incidence of complications. Therefore, if we can further optimize the shape of the hook plate and select the hook plate with different sizes, depths, and angles according to individualized patients, it will help reduce the occurrence of complications and improve the clinical effect.

The study had several limitations. First, this was a retrospective study. Second, it was an insufficient statistical power because of the non-controlled study. Despite having no comparison to other approaches, the presented data provided evidence that hook plate fixation achieved good clinical outcomes and fewer complications in the treatment of acromioclavicular dislocation.

## Conclusion

In conclusion, we demonstrated that hook plate fixation was a simple and viable treatment approach, and achieved good clinical outcomes in the treatment of high-grade ACD involving V. Meanwhile, we should note that some complications of hook plate fixation should not be ignored.

## Data Availability

Data and materials were available from the corresponding author.

## References

[1] Martetschläger F, Kraus N, Scheibel M, Streich J, Venjakob A, Maier D (2019) The diagnosis and treatment of acute dislocation of the acromioclavicular joint. Deutsches Aerzteblatt Online. 10.3238/arztebl.2019.0089 10.3238/arztebl.2019.0089 PMC6435864 30892184

[2] Pan X, Ry Lv, Mg Lv, Dg Z (2020). TightRope vs clavicular hook plate for Rockwood III–V acromioclavicular dislocations: a meta-analysis. Orthop Surg.

[3] Nolte PC, Lacheta L, Dekker TJ, Elrick BP, Millett PJ (2020). Optimal management of acromioclavicular dislocation: current perspectives. Orthop Res Rev.

[4] Phadke A, Bakti N, Bawale R, Singh B (2019). Current concepts in management of ACJ injuries. J Clin Orthop Trauma.

[5] Tamaoki MJS, Lenza M, Matsunaga FT, Belloti JC, Matsumoto MH, Faloppa F (2019) Surgical versus conservative interventions for treating acromioclavicular dislocation of the shoulder in adults. Cochrane Database Syst Rev. 10.1002/14651858.CD007429.pub3 10.1002/14651858.CD007429.pub3 PMC6788812 31604007

[6] Frank RM, Cotter EJ, Leroux TS, Romeo AA (2019). Acromioclavicular joint injuries: evidence-based treatment. J Am Acad Orthop Surg.

[7] Xu D, Luo P, Chen J, Ji L, Yin L, Wang W, Zhu J (2017). Outcomes of surgery for acromioclavicular joint dislocation using different angled hook plates: a prospective study. Int Orthop.

[8] Jeong JY, Chun Y-M (2020). Treatment of acute high-grade acromioclavicular joint dislocation. Clin Shoulder Elb.

[9] Takase K, Hata Y, Morisawa Y, Goto M, Tanaka S, Hamada J, Hayashida K, Fujii Y, Morihara T, Yamamoto N, Inui H, Shiozaki H (2021). Treatment of acromioclavicular joint separations in Japan: a survey. JSES Int.

[10] Li G, Liu T, Shao X, Liu Z, Duan J, Akileh R, Cao S, Jin D (2018). Fifteen-degree clavicular hook plate achieves better clinical outcomes in the treatment of acromioclavicular joint dislocation. J Int Med Res.

[11] Lin HY, Wong PK, Ho WP, Chuang TY, Liao YS, Wong CC (2014). Clavicular hook plate may induce subacromial shoulder impingement and rotator cuff lesion–dynamic sonographic evaluation. J Orthop Surg Res.

[12] Yoon JP, Lee B-J, Nam SJ, Chung SW, Jeong W-J, Min W-K, Oh JH (2015). Comparison of results between hook plate fixation and ligament reconstruction for acute unstable acromioclavicular joint dislocation. Clin Orthop Surg.

[13] McConnell AJ, Yoo DJ, Zdero R, Schemitsch EH, McKee MD (2007). Methods of operative fixation of the acromio-clavicular joint: a biomechanical comparison. J Orthop Trauma.

[14] Williams GR, Nguyen VD, Jr CAR (1989) Classification and radiographic analysis of acromioclavicular dislocations. Appl Radiol 18:29–34

[15] Constant CR, Murley AH (1987) A clinical method of functional assessment of the shoulder. Clin Orthop Relat Res 160–164 3791738

[16] Ellman H, Hanker G, Bayer M (1986). Repair of the rotator cuff. End-result study of factors influencing reconstruction. J Bone Joint Surg Am.

[17] Bannister GC, Wallace WA, Stableforth PG, Hutson MA (1989). The management of acute acromioclavicular dislocation. A randomised prospective controlled trial. J Bone Joint Surg Br.

[18] Modi CS, Beazley J, Zywiel MG, Lawrence TM, Veillette CJ (2013). Controversies relating to the management of acromioclavicular joint dislocations. Bone Joint J.

[19] Domos P, Sim F, Dunne M, White A (2017). Current practice in the management of Rockwood type III acromioclavicular joint dislocations—National survey. J Orthop Surg.

[20] Arirachakaran A, Boonard M, Piyapittayanun P, Kanchanatawan W, Chaijenkij K, Prommahachai A, Kongtharvonskul J (2017). Post-operative outcomes and complications of suspensory loop fixation device versus hook plate in acute unstable acromioclavicular joint dislocation: a systematic review and meta-analysis. J Orthop Traumatol.

[21] Allemann F, Halvachizadeh S, Waldburger M, Schaefer F, Pothmann C, Pape HC, Rauer T (2019) Different treatment strategies for acromioclavicular dislocation injuries: a nationwide survey on open/minimally invasive and arthroscopic concepts. Eur J Med Res 24. 10.1186/s40001-019-0376-7 10.1186/s40001-019-0376-7 PMC6431035 30904018

[22] Taleb H, Afshar A, Shariyate MJ, Tabrizi A (2019). Comparison of short-term clinical outcomes of hook plate and continuous loop double Endobutton fixations in acute acromioclavicular joint dislocation. Arch Bone Jt Surg.

[23] Jensen G, Katthagen JC, Alvarado LE, Lill H, Voigt C (2012). Has the arthroscopically assisted reduction of acute AC joint separations with the double tight-rope technique advantages over the clavicular hook plate fixation?. Knee Surg Sports Traumatol Arthrosc.

[24] Bin AbdRazak HR, Yeo E-MN, Yeo W, Lie T-TD (2017). Short-term outcomes of arthroscopic TightRope® fixation are better than hook plate fixation in acute unstable acromioclavicular joint dislocations. Eur J Orthop Surg Traumatol.

[25] Huang Y-C, Yang S-W, Chen C-Y, Lin K-C, Renn J-H (2018) Single coracoclavicular suture fixation with Mersilene tape versus hook plate in the treatment of acute type V acromioclavicular dislocation: a retrospective analysis. J Orthop Surg Res 13. 10.1186/s13018-018-0831-0 10.1186/s13018-018-0831-0 PMC5956760 29769141

[26] Clavert P, Meyer A, Boyer P, Gastaud O, Barth J, Duparc F (2015). Complication rates and types of failure after arthroscopic acute acromioclavicular dislocation fixation. Prospective multicenter study of 116 cases. Orthop Traumatol Surg Res.

[27] Kienast B, Thietje R, Queitsch C, Gille J, Schulz AP, Meiners J (2011). Mid-term results after operative treatment of rockwood grade III-V acromioclavicular joint dislocations with an AC-hook-plate. Eur J Med Res.

[28] Kumar N, Sharma V (2015). Hook plate fixation for acute acromioclavicular dislocations without coracoclavicular ligament reconstruction: a functional outcome study in military personnel. Strategies Trauma Limb Reconstr.

[29] Liu C-T, Yang T-F (2020) Hook plate with or without coracoclavicular ligament augmentation in the treatment of acute acromioclavicular separation. BMC Musculoskelet Disord 21. 10.1186/s12891-020-03726-z 10.1186/s12891-020-03726-z PMC7585224 33097023

[30] Di Francesco A, Zoccali C, Colafarina O, Pizzoferrato R, Flamini S (2012). The use of hook plate in type III and V acromio-clavicular Rockwood dislocations: clinical and radiological midterm results and MRI evaluation in 42 patients. Injury.

[31] Seo J-B, Kim S-J, Ham H-J, Yoo J-S (2020). Comparison between hook plate fixation with and without coracoclavicular ligament suture for acute acromioclavicular joint dislocations. J Orthop Surg.

[32] Chen Y-T, Wu K-T, Jhan S-W, Hsu S-L, Liu H-C, Wang C-J, Ko J-Y, Chou W-Y (2021) Is coracoclavicular reconstruction necessary in hook plate fixation for acute unstable acromioclavicular dislocation? BMC Musculoskelet Disord 22. 10.1186/s12891-021-03978-3 10.1186/s12891-021-03978-3 PMC7849128 33522921

[33] Masionis P, Bobina R, Ryliskis S (2020). The relationship between the clinical and radiological findings and the outcomes of early surgical treatment after Tossy Type III acromioclavicular joint dislocation. Cureus.

[34] Lee C-H, Shih C-M, Huang K-C, Chen K-H, Hung L-K, Su K-C (2016). Biomechanical analysis of implanted clavicle hook plates with different implant depths and materials in the acromioclavicular Joint: a finite element analysis study. Artif Organs.

[35] Yoon JP, Lee YS, Song GS, Oh JH (2016). Morphological analysis of acromion and hook plate for the fixation of acromioclavicular joint dislocation. Knee Surg Sports Traumatol Arthrosc.

[36] Qiao R, Yang J, Zhang K, Song Z (2021) To explore the reasonable selection of clavicular hook plate to reduce the occurrence of subacromial impingement syndrome after operation. J Orthop Surg Res 16. 10.1186/s13018-021-02325-5 10.1186/s13018-021-02325-5 PMC7944884 33750451

